# beeRapp: an R shiny app for automated high-throughput explorative analysis of multivariate behavioral data

**DOI:** 10.1093/bioadv/vbac082

**Published:** 2022-11-07

**Authors:** Anne Marie Busch, Irina Kovlyagina, Beat Lutz, Hristo Todorov, Susanne Gerber

**Affiliations:** Institute of Human Genetics, University Medical Center of the Johannes Gutenberg University Mainz, Mainz 55128, Germany; Institute of Physiological Chemistry, University Medical Center of the Johannes Gutenberg University Mainz, Mainz 55128, Germany; Institute of Physiological Chemistry, University Medical Center of the Johannes Gutenberg University Mainz, Mainz 55128, Germany; Leibniz Institute for Resilience Research (LIR), Mainz 55122, Germany; Institute of Human Genetics, University Medical Center of the Johannes Gutenberg University Mainz, Mainz 55128, Germany; Institute of Human Genetics, University Medical Center of the Johannes Gutenberg University Mainz, Mainz 55128, Germany

## Abstract

**Summary:**

Animal behavioral studies typically generate high-dimensional datasets consisting of multiple correlated outcome measures across distinct or related behavioral domains. Here, we introduce the BEhavioral Explorative analysis R shiny APP (beeRapp) that facilitates explorative and inferential analysis of behavioral data in a high-throughput fashion. By employing an intuitive and user-friendly graphical user interface, beeRapp empowers behavioral scientists without programming and data science expertise to perform clustering, dimensionality reduction, correlational and inferential statistics and produce up to thousands of high-quality output plots visualizing results in a standardized and automated way.

**Availability and implementation:**

The code and data underlying this article are available at https://github.com/anmabu/beeRapp.

## 1 Introduction

Animal behavioral models, especially in rodents, are widely used in basic and translational research to study neuropsychiatric, neurodevelopmental and neurodegenerative disorders (Le *et al.*, 2016; Nestler and Hyman, 2010; Silverman and Ellegood, 2018) and the impact of traumatic events on the central nervous system (Shultz *et al.*, 2020) or in drug discovery research (Hånell and Marklund, 2014). To this end, various tests are applied to assess anxiety- and depressive-like behavior, sociability, learning, memory and sensory–motor function (Sousa *et al.*, 2006). Typically, in each test, several parameters are quantified. Afterward, a multidimensional dataset, with up to hundreds of individual variables, can be assembled by combining all test results. Importantly, outcome measures within the same test or across related behavioral domains are often not independent of each other; thus, investigating the multivariate correlation patterns is essential to ensure the validity of the behavioral paradigm. From a data science viewpoint, challenges in analyzing such behavioral datasets arise due to the necessity to apply multivariate statistical methods as well as to handle a vast number of potential univariate comparisons. While commercial software like GraphPad Prism and SPSS offer a wide variety of sophisticated statistical tests, these programs are not open source and cannot facilitate automated high-throughput analysis. The statistical language R overcomes both issues. However, the lack of a graphical user interface (GUI) implies advanced programming skills, making this an unattractive option for behavioral researchers without data science experience. Here, we present BEhavioral Explorative analysis R shiny APP (beeRapp), which combines a wide range of explorative and inferential statistical techniques and automates results visualization with a GUI implemented in R Shiny. The GUI circumvents the need to write scripts in R, making multivariate explorative behavioral data analysis accessible to scientists with a primary focus on experimental research.

## 2 Implementation

beeRapp was implemented in R version 4.1.3 and tested using R version 4.2.1, however installing, and using it is not limited to a specific R version. The source code can be downloaded from the project’s website (https://github.com/anmabu/beeRapp) where users can find instructions for installation, additional information, example data and a detailed vignette of how to use the app. The input for beeRapp includes a table containing the behavioral outcome measures; a table with meta data such as group assignment, genotype or phenotype and a table with more extensive descriptions of the behavioral measurements used for automatic labeling of the axes on the output figures. The example dataset on the project’s website demonstrates the formatting and naming conventions of the input tables necessary for submitting the data to beeRapp.

**Fig. 1.**
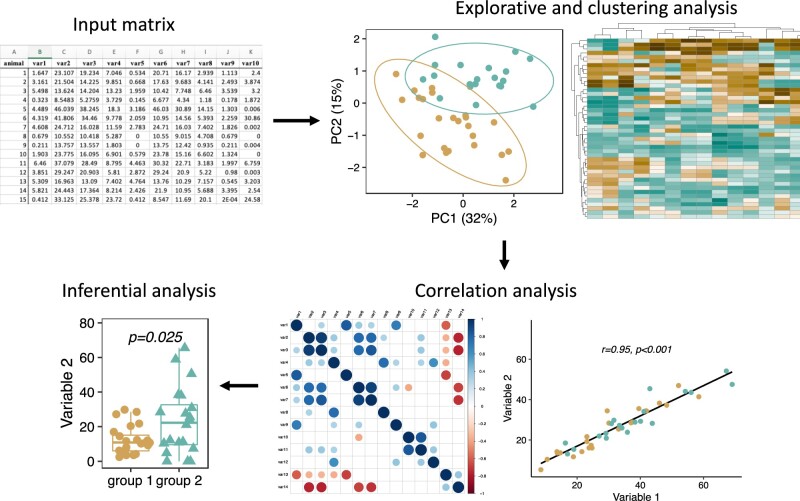
Overview of beeRapp’s analysis workflow. The input behavioral data matrix can be analyzed using different modules, including clustering, dimensionality reduction, heatmap representation, multivariate and pairwise correlational analysis and inferential statistics. Each module can be executed independently. Additionally, clustering results can be integrated with subsequent analyses, such as pairwise statistical comparisons

Figure 1 provides an overview of the different analysis modules and representative result images obtained with beeRapp.

### 2.1 Explorative analysis

This module performs a principal component analysis (PCA) (Todorov *et al.*, 2018) of the input behavioral data, which is useful for investigating general trends or scanning for multivariate outliers. In the absence of missing data, PCA is performed on the centered and scaled data matrix using the *prcomp* function. If missing data are present in the matrix, the analysis utilizes the *pca* function from the *mixOmics* package (Rohart *et al.*, 2017). The input matrix can also be visualized with a heatmap produced with the *pheatmap* package (Kolde, 2019). Per default, data are scaled to zero mean and unit variance to account for different measurement scales. Both PCA and heatmap analysis allow the user to include a grouping variable that can be visualized on the output plots.

### 2.2 Clustering analysis

Behavioral researchers are often interested in identifying subgroups of animals in the investigated populations. beeRapp’s clustering module enables this by providing the option to perform a *k*-means, a Gaussian mixture model (GMM) or hierarchical clustering using any variable(s) from the input matrix. By default, at least two clusters are produced. The *k*-means option utilizes the standard implementation of the *stats* package, whereas GMM is performed with the *mclust* package (Scrucca *et al.*, 2016). Hierarchical clustering is achieved with the *hclust* function from the *stats* package with the default implementation which uses the complete linkage method on the Euclidean distance matrix. Subsequently, the *cutree* function is applied to produce the desired number of clusters. Clustering is visualized with a scatter plot showing either the original variables if up to two variables are used or a PCA representation based on the input variables when more than or equal to three measures are used for clustering. Ninety-five percent confidence ellipses are drawn around each cluster. Apart from specifying the number of clusters based on theoretical considerations themselves (e.g. distinguishing between populations of high- and low-freezing animals or stress-affected versus unaffected), users can also let beeRapp elucidate the optimal number of clusters. For GMM clustering, this is facilitated with the build-in *clustCombiOptim* function from the *mclust* package. This method automates optimal cluster selection by first fitting a mixture model with the number of components chosen based on the Bayesian information criterion and then combining these components hierarchically based on an entropy criterion. The optimal number of clusters is selected as the ‘breakpoint’ on a linear fit of the rescaled entropy against the number of clusters using a piecewise linear regression [for a detailed description of the method confer Baudry *et al.* (2010)]. For *k*-means and hierarchical clustering, the relevant number of clusters is obtained with the *NbClust* package (Charrad *et al.*, 2014). To this end, 30 different indices are calculated by the package and then a majority vote is taken for the optimal number of clusters. We do this for a range of two to five clusters as we believe that a higher number of clusters is unrealistic for animal behavior data. The dissimilarity matrix is calculated using a Euclidean distance and we use the ‘complete’ and ‘*k*-means’ methods for hierarchical and *k*-means clustering, respectively.

One common issue that GMM and *k*-means clustering face is that both algorithms cannot handle missing data. We recommend that users do not include variables in the clustering procedures if the percentage of missing values is high (e.g. >20%). However, we also use the *Hmisc* package to impute missing values based on bootstrap predictive mean matching with five multiple imputations as implemented in the *aregImpute* function.

### 2.3 Correlation analysis

The relationships between different behavioral measurements can be visualized with a correlation matrix showing all Pearson or Spearman correlations below a chosen significance threshold (*P* < 0.05 per default). Correlation matrices are visualized with the *corrplot* package (Wei and Simko, 2021). Furthermore, pairwise scatter plots showing the trend line from a linear regression analysis, the correlation coefficients and the corresponding *P*-value can be produced for each possible combination of variables using the *cor.test* function from base R. Data points can be colored according to a group assignment provided in the meta data file or obtained via the clustering module. Scatter plots can be exported as a pdf or PowerPoint file, providing a compact solution to save and view multiple individual (up to thousands) pairwise correlation plots.

### 2.4 Pairwise comparisons

This module allows the user to compare two groups statistically using a non-parametric Wilcoxon rank sum test (Wilcoxon, 1945), a parametric *t*-test or a permutation test. The grouping variable can be provided in the meta data table or obtained using the clustering module. If the *t*-test option is selected, the app automatically performs a variance homogeneity test using the *var.test* function from base R, which compares the variance ratio of the two groups against an *F* distribution. When the variance ratio differs significantly from 1 indicating variance inhomogeneity (*P *≤* *0.05), the app utilizes a Welch’s *t*-test (Welch, 1947). Finally, pairwise comparisons can be facilitated with a more general permutation-based test for independence as implemented in the *coin* package (Hothorn *et al.*, 2008). In this case, the test statistic is compared against an asymptotic null distribution generated using a randomized quasi-Monte Carlo method. A permutation-based test has the advantage that no assumptions about the distribution of the variables are made. Furthermore, the user also has the option to automatically screen for outliers and remove them from the analysis, which is recommended when a parametric *t*-test is performed as outliers can significantly impact the results of parametric tests. Outliers are detected by transforming the original values to *z*-scores and removing values with an absolute *z*-score exceeding 3.29, *P* < 0.001, two-sided *z*-test, as recommended (Tabachnik and Fidel, 2014). Depending on the selected significance threshold, either all plots are produced or only plots for which the *P*-value is below the chosen cut-off. Data are visualized with box and whiskers plots and figures can be exported either as a pdf or a PowerPoint file.

### 2.5 Generation of analysis reports

**Fig. 2.**
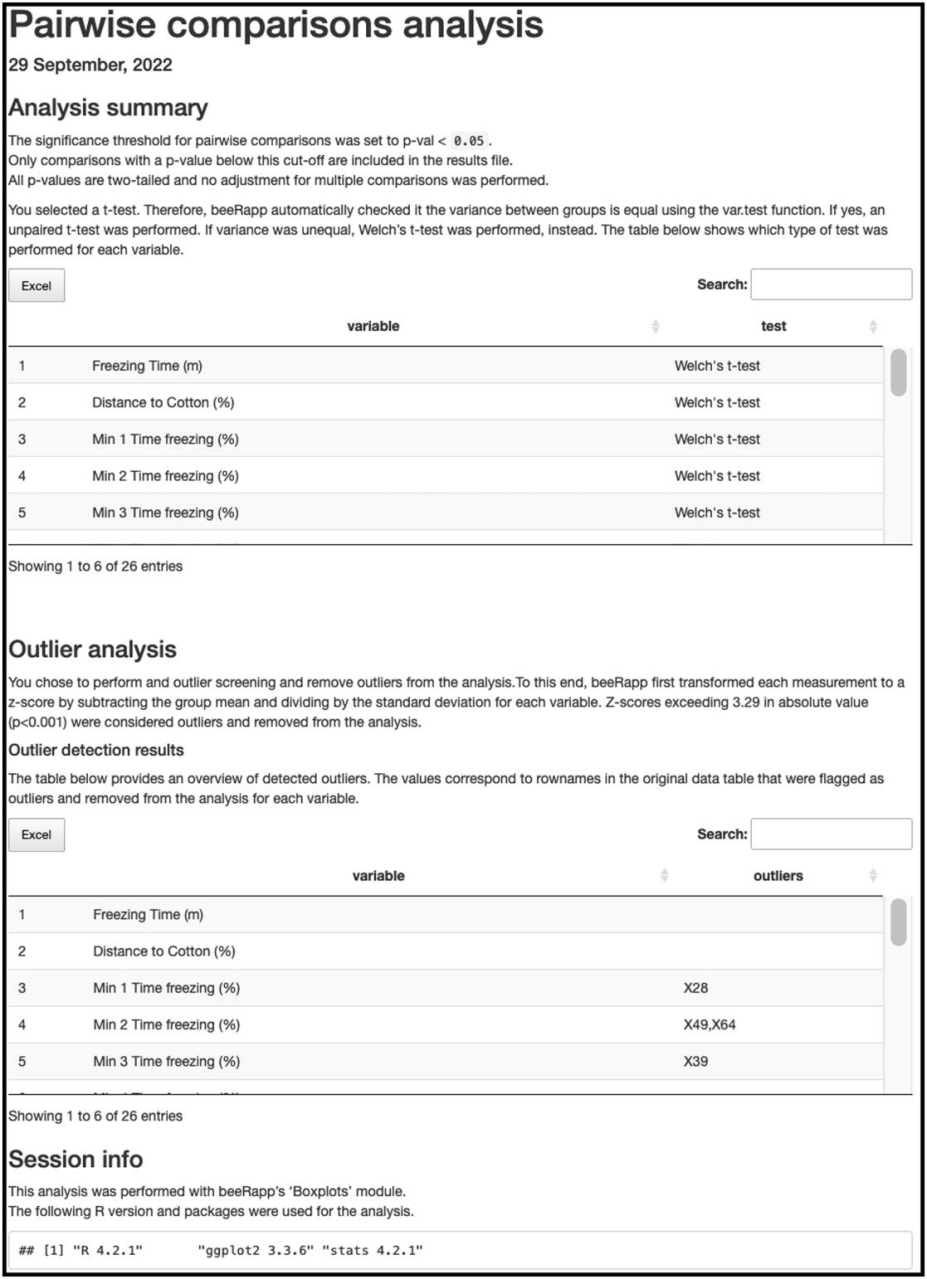
A representative analysis report file. The example html document contains details for the pairwise comparisons analysis performed by beeRapp including selected settings and the R packages used for the analysis

Most modules in beeRapp contain several options for performing a given analysis, for example, different clustering algorithms or statistical tests for pairwise comparisons. To guarantee reproducibility of results, the user has the option to download an analysis report. These html files contain a summary of the analytical methods with the specific settings applied to the particular data analysis. Furthermore, technical details such as R packages and versions used are included in the report. A representative report file is shown in Fig. 2.

## 3 Conclusion

beeRapp leverages the vast explorative and inferential statistical analysis capacity of R and the user-friendliness of R shiny apps, allowing behavioral researchers to explore complex relationships and patterns in the generated datasets. We acknowledge that other existing R shiny apps such as ExPanD (https://jgassen.shinyapps.io/expand/) or Statsomat (https://statsomat.com/) already offer powerful tools for interactive data exploration, descriptive statistics, dimensionality reduction and correlational analysis. Despite this, beeRapp still manages to provide unique features (e.g. heatmap visualization, clustering analysis and automated generation of multiple, customizable, high-resolution and downloadable figures that can be integrated readily in scientific publications). Rather than just being a collection of tools, beeRapp provides an analysis workflow co-developed with and tailored to the needs of experimental behavioral scientists which also sets it apart from existing solutions. Since the app is installed and run locally and not hosted on a web server, there is no limitation to the size of the input dataset and number of variables. Starting beeRapp from GitHub directly using the command *shiny::runGitHub(‘beeRapp’, ‘anmabu’)* ensures that users always work with the most current version without the need to reinstall the app. While fitting specific more elaborate models still requires data science proficiency, beeRapp nevertheless provides experimental scientists a substantial level of independence to perform a wide variety of sophisticated analyses without the explicit support from bioinformatics and biostatistics experts.
